# Atrazine, triketone herbicides, and their degradation products in sediment, soil and surface water samples in Poland

**DOI:** 10.1007/s11356-016-7798-3

**Published:** 2016-10-14

**Authors:** Hanna Barchanska, Marcin Sajdak, Kornelia Szczypka, Angelika Swientek, Martyna Tworek, Magdalena Kurek

**Affiliations:** 1 Department of Inorganic, Analytical Chemistry and Electrochemistry, Faculty of Chemistry, Silesian University of Technology, B. Krzywoustego 6 Str, 44-100 Gliwice, Poland; 2 Institute for Chemical Processing of Coal, 1 Zamkowa St, 41-803 Zabrze, Poland

**Keywords:** Triketones, Atrazine, Herbicide degradation products, Soil, Sediment, Surface water

## Abstract

The aim of this study was to monitor the sediment, soil and surface water contamination with selected popular triketone herbicides (mesotrione (MES) and sulcotrione(SUL)), atrazine (ATR) classified as a possible carcinogen and endocrine disrupting chemical, as well as their degradation products, in Silesia (Poland). Seventeen sediment samples, 24 soil samples, and 64 surface water samples collected in 2014 were studied. After solid-liquid extraction (SLE) and solid phase extraction (SPE), analytes were determined by high-performance liquid chromatography (HPLC) with diode array detection (DAD). Ten years after the withdrawal from the use, ATR was not detected in any of the collected samples; however, its degradation products are still present in 41 % of sediment, 71 % of soil, and 8 % of surface water samples. SUL was determined in 85 % of soil samples; its degradation product (2-chloro-4-(methylosulfonyl) benzoic acid (CMBA)) was present in 43 % of soil samples. In 17 % of sediment samples, CMBA was detected. Triketones were detected occasionally in surface water samples. The chemometric analysis (clustering analysis (CA), single-factor analysis of variance (ANOVA), N-Way ANOVA) was applied to find relations between selected soil and sediment parameters and herbicides concentration. In neither of the studied cases a statistically significant relationship between the concentrations of examined herbicides, their degradation products and soil parameters (organic carbon (OC), pH) was observed.

**Electronic supplementary material:**

The online version of this article (doi:10.1007/s11356-016-7798-3) contains supplementary material, which is available to authorized users.

## Introduction

Since their discovery in the 1940s, pesticides have greatly contributed to improving the yield and quality of crops and to ensuring their production. Atrazine ((2-chloro-4-ethylamino-6-isopropylamino-1,3,5-triazine, ATR), a triazine compound, has been frequently used (about 30,000 tons annually) as a herbicide for maize crops (Hase et al. 2008). ATR accumulate in leaves and meristems, where, in sensitive plant species, it inhibits the Hill reaction by blocking photosynthesis by binding with tyrosinase, the enzyme responsible for the oxidation of polyphenols to quinones, which, in consequence, lead to annihilation of weeds (Mou et al. 2011). ATR inhibits the growth of weeds and algae by interfering with the normal function of photosynthesis. The widespread and long-term use of ATR resulted in its high residue levels in soil, which further causes the surface and groundwater contamination via rain runoff and leakage (Ji et al. 2015).

ATR is degraded in the environment to a range of degradation products: hydroxyatrazine (2-hydroxy-4-ethylamino-6-isopropylamino-1,3,5-triazine, HA); deethylatrazine (2-chloro-4-amino-6-ethylamino-1,3,5-triazine, DEA); deisopropylatrazine (2-chloro-4-ethylamino-6-amino-1,3,5-triazine, DIA) and desethyldesisopropylatrazine (2-chloro-4,6-diamino-1,3,5-triazine, DEDIA). These compounds, similar to parent one, are persistent in the environment and toxic (potent endocrine disrupters) (Ghanem et al. 2008; Hu and Cheng 2013).

Due to ATR high toxicity (possible carcinogen and endocrine disrupting chemical to numerous organisms), persistence and ability to transfer in the environment (Roustan et al. 2014; Yixin et al. 2014), it was banned in several European countries in 2003 (still widely used in the USA and another region of the world). As a replacement, new selective herbicides have been developed. Among them, the triketone class of herbicides, mainly mesotrione and sulcotrione play the most important role (Calvayrac et al. 2012; Chaabane et al. 2008; Jović et al. 2013; Tawk et al. 2015).

Synthetic β-triketone herbicides inhibit plant 4-hydroxyphenylpyruvate dioxygenase (HPPD) activity that leads to decrease in the pigment production, leaves bleaching and finally, death of the plant (Jović et al. 2013; Owens et al. 2013). They are used to control the majority of annual broadleaf weeds, with limited activity on grasses. Among the group of triketone herbicides, the most frequently used are mesotrione (2-(4-methylsulfonyl-2-nitrobenzoyl)-1, 3-cyclohexanedione, MES) and sulcotrione (2-[2-chloro-4-(methylsulfonyl)benzoyl]-1,3-cyclohexanedione, SUL).

MES was developed by Syngenta Crop Protection and registered in Europe in 2000, whereas SUL was introduced by Zeneca Ag Products (now Syngenta Crop Protection) and first registered for use in France in 1993. Both MES and SUL have acidic properties (pKa of around 3), which are determinant for their environmental behaviour. In the environment, MES is degraded into two main by-products: 4-(methylsulfonyl)-2-nitrobenzoic acid (MNBA) and 2-amino-4-(methylsulfonyl)benzoic acid (AMBA), whereas 2-chloro-4-(methylosulfonyl) benzoic acid (CMBA) and 1,3-cyclohexanedione (CHD) are the degradation products of SUL (Trivella et al. 2015).

According to numerous literature reports (Barchanska et al. 2014; Bardot et al. 2015; Crouzet et al. 2010, 2013; Joly et al. 2013), triketones and their degradation products negatively influence microorganisms and aquatic plants. MES exhibits moderate retention capacity in different soils and may be leached to surface water (Chaabane et al. 2008; Dyson et al. 2002). Moreover, MES is also classified by the EEC as toxicologically dangerous for the environment (Batisson et al. 2009). MES can persist in the soil up to 32 days after application depending on the environmental conditions and type of soil (Dyson et al. 2002). Its residues affect several sensitive crops, such as snap beans, pickling cucumber, cabbage and pepper (Yu et al. 2015). Moreover, it was shown that the AMBA had a higher toxicity than the parent compound (Barchanska et al. 2014; Bonnet et al. 2008).

Although SUL degradation products had no herbicide activity, they present toxicity towards unicellular organisms different of sulcotrione toxicity (Ter Halle et al. 2009; Wiszniowski et al. 2009). According to Goujon et al. (2014), SUL possesses genotoxic properties against *Allium cepa* L. The detailed physicochemical parameters of all above-mentioned herbicides and their degradation products are placed in Table 1.

**Table 1:**
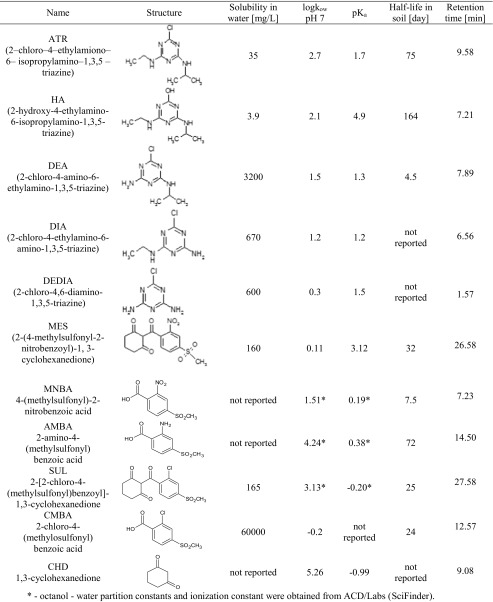
Characteristic of investigated herbicides and their degradation products

sitem.herts.ac.uk/aeru/iupac/index.htm (access: 22.10.2015)

*Octanol-water partition constants and ionization constant were obtained from ACD/Labs (SciFinder)

During the agricultural treatments, pesticides could be directly reached by the cultures or sorbed into the soil particles. The behaviour of pesticides in soils is governed by a variety of complex dynamic physical, chemical and biological processes, including; volatilization, sorption–desorption, chemical and biological degradation, uptake by plants, run-off and leaching (Alekseeva et al. 2014). All these processes depend on the physicochemical properties of pesticide, environmental conditions, microorganisms and properties of soil, and they directly control the transport of pesticides within the soil and their transfer from the soil to water and sediments. The water contamination with pesticides is constantly monitored by the European Union. It has established a number of regulations and directives, such as the Directive 2000/60/EC, which defines the framework for the management and the restoration of the status of surface and superficial waters for 2015 (European Union 2000, 2009; Rocaboy-Faquet et al. 2014). The maximum allowable pesticide concentration in drinking water cannot exceed 0.1 μg/L.

From the surface waters, pesticides are deposited into bottom sediments. Pesticides sorbed on sediments are hardly bioavailable; however, they may have an influence on benthos organisms. As a result of environmental condition changes (increased temperature, pH, etc.) or floods, the pesticides accumulated in the sediments are in an uncontrolled way released back into the environment.

Therefore, it is necessary to monitor the concentration of pesticide residues in soil, water and sediments as well as aquatic organisms, since from these matrices, pesticides are transferred into the air and food of plant origin, which constitutes a direct threat to human health.

According to our best knowledge, there is a scarce current data concerning the monitoring of ATR in European environment (Price et al. 2006; Caquet et al. 2013; Farlin et al. 2013, Ouyang et al. 2016a). The main attention is paid to degradation and transformation of ATR during water treatment processes (Baranda et al. 2012; Lekkerkerker-Teunissen et al. 2012; Yixin et al. 2014; Cheng et al. 2016), its toxicity (Roustan et al. 2014) or sorption behaviour in soil (Prado et al. 2014; Nachimuthu et al. 2016; Ouyang et al. 2016b). Similarly, monitoring information concerning triketone herbicides and their degradation products (Alferness and Wiebe 2002; Freitas et al. 2004; Moschet et al. 2014) are lacking.

In conclusion, according to our literature review as well as studies conducted by Farlin et al. (2013) the historical and the spatial monitoring of the pesticide residues after several half-life cycles has seldom been conducted. Such investigations are of special importance since pesticides, that are slowly degraded in soil, are potential markers that could prove useful for a number aspects pertaining to pesticide fate modelling. Therefore, the objective of the present research was to conduct the monitoring studies concerning the presence of ATR, MES and SUL, and their degradation products in sediments (17 samples), soil (22 samples) and surface water (64 samples) collected in 2014 in the Silesia region (Poland).

## Material and methods

### Apparatus and chemicals

Herbicide standards and their degradation products were supplied by Sigma-Aldrich, Germany (ATR, DIA, DEA, DEDIA, HA, MES, SUL, TEMB and TEMB MET); Santa Cruz Biotechnology, Germany (AMBA and MNBA); and Dr. Ehrenstorfer Quality, Germany (CHD and CMBA). Stock standard solutions for each compound were prepared in methanol at 1 mg/mL and stored in glass vials at 4 °C in the dark. Acetonitrile, trifluoroacetic acid (TFA), and water (all high-performance liquid chromatography (HPLC) grade) were from Merck, Germany. Acetic acid (glacial), acetonitrile, dichloromethane, ethyl acetate, methanol, and hydrochloric acid conc. (all analytical grade) were purchased from Stanlab, Poland.

The chromatographic system used (Merck Hitachi, Germany) consisted of a Hitachi LaChrom Elite L-2130 pump and Hitachi LaChrom Elite L-2455 photodiode array detector (DAD). The stationary-phase column was LiChroCART Purospher RP-18e (125 × 3 mm, 5 μm, Merck).

For solid phase extraction (SPE), the following sorbents were applied: OASIS® HLB (500 mg, 6 mL; Waters, Milford, USA), SDB (500 mg, 3 mL) and C18 (500 mg, 3 mL). These sorbents were used on a 12-fold vacuum extraction box (BAKERBOND® SPE J.T. Baker, Philipsburg, USA). Nylon membrane filters were also obtained from J.T. Baker. Chemometric analysis was performed using MATLAB R2015b software.

### Samples

Seventeen sediment, 24 soil and 64 surface water samples, collected in 2014 were studied.

River sediment samples were collected according to the standards ISO 5667-15:2009 (2009) in September and October 2014. From Kłodnica River, sediment samples (nos. 1–7) were taken along the river from depths 0.3 m and 1.2 m.

Soil samples were collected according to ISO 10381-4:2007 (2007) in July and August 2014. Before all experiments, stones and plant fragments were removed, the soils and sediments were air-dried, until the constant mass and sieved through 0.2 mm mesh.

Water samples were collected periodically every month (January 2014–September 2014). Raw water samples, collected according to PN-ISO 5667-4: 2003 and PN-ISO 5667-6: 2003, were stored with added acid (pH 2.5) in the dark at 4 °C. Samples were filtered at room temperature through a Buchner funnel and then nylon membrane filters (0.2 μm).

**Fig. 1:**
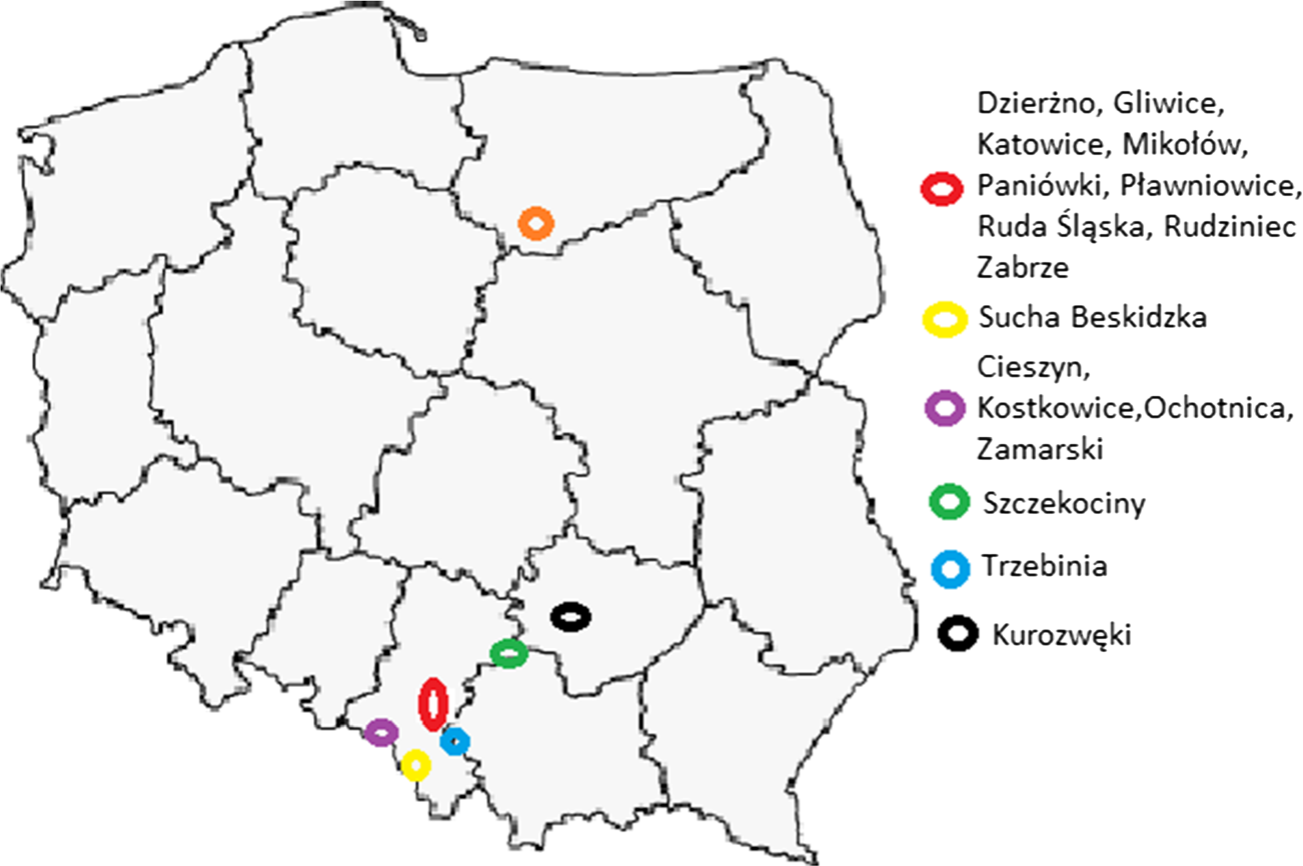
Samples origin

The samples were collected mainly in the Silesian Upland (Poland) from agricultural, forest and industrial regions, as well as in agricultural areas of central and south Poland. During the whole period of sampling, the level of atmospheric precipitation was monitored in the investigated regions. The detailed information about the sample origin is placed in Fig. 1 and Table 2.

**Table 2:** Samples origin—general information

Sediment samples			
No.	Date	Place/river	Remarks
1	September 2014	Katowice Giszowiec-Kłodnica	forest, marshy area
2	September 2014	Gliwice Łabędy-Kłodnica	water treatment plant, maize and potatoes cultivations
3	September 2014	Ruda Śląska-Kłodnica	potatoes, maize and corn cultivations
4	September 2014	Gliwice-Kłodnica	recreational area
5	September 2014	Zabrze Makoszowy-Kłodnica	potatoes cultivation
6	September 2014	Zabrze Makoszowy-Kłodnica	wheat, rape, rye cultivations
7	September 2014	Katowice-Kłodnica	forest, marshy area
8	October 2014	Ochotnica Dolna-Ochotniczanka	meadow
9	October 2014	Zamarski- Lutnia	meadow
10	October 2014	Kostkowice-stream	meadow
11	October 2014	Cieszyn-Olza	industrialized area, water treatment plant
12	October 2014	Cieszyn-Bobrówka	industrialized area power plant
13	October 2014	Cieszyn-Młynówka	industrialized area power plant
14	October 2014	Cieszyn-Przykopa	wheat cultivation
15	September 2014	Tychy-Gostyń	potatoes cultivation
16	September 2014	Tychy-Mleczna	maize cultivation
17	September 2014	Katowice-Rawa	water treatment plant
Soil samples			
No	Date	Place	Remarks
18–21	July 2014	Szczekociny I-IV	potatoes cultivation
22–24	August 2014	Sucha Beskidzka I-III	meadow, potatoes, cucumber and cultivations
25	August 2014	Katowice Kostuchna	wheat cultivation, forest
26–28	August 2014	Tychy Wilkowyje I-III	wheat cultivation
29	August 2014	Katowice Kostuchna	meadow
30	August 2014	Paniówki	maize cultivation
31	August 2014	Mikołów	maize cultivation
32	August 2014	Ruda Śląska	forest
33–37	August 2014	Kurozwęki I-V	potatoes cultivation
38	October 2014	Kozłowo	maize cultivation
39	October 2014	Rudziniec	maize cultivation
40	August 2014	Trzebinia	maize cultivation
41	August 2014	Gliwice	potatoes cultivation
Water samples			
42	January–September (monthly) 2014	Pławniowice Lake	artificial lake, surrounded by agricultural area (the cultivation of cereals)
43	January–September (monthly) 2014	Dzierżno Lake	artificial lake, surrounded by farmland (the cultivation of cereals), powered by the waters of Kłodnica river
44	January–September (monthly) 2014	Rudziniec/breeding pond	natural lake, surrounded by agricultural area (the cultivation of cereals)
45	January–September (monthly) 2014	Dziergowice/Dziergowice Lake	artificial lake, surrounded by farmland (the cultivation of cereals), powered by the waters of Bierawka river
46	January–September (monthly) 2014	Rudziniec/Kłodnica River	river flowing through the Silesian Upland, place of sampling—agricultural area
47	January–September (monthly) 2014	Rudziniec/drainage ditch	a drainage ditch in agricultural area
48	January–September (monthly) 2014	Rudziniec/ pond in forest	natural lake, surrounded by forest

### Analytical procedures

#### Triazines determination

From soil and water samples, atrazine and its derivative was extracted according to Barchanska et al. (2012). Ten grams of soil was extracted with 30 mL of the mixture ACN:0.1 M HCl (9:1 *v*/*v*); after filtering, the extract was shaken with dichloromethane (2 × 5 mL). After phase separation, the organic extract was evaporated to dryness, dissolved in 5 mL of 0.1 M HCl and concentrated by solid phase extraction (SPE). Silica gel modified with octadecyl groups (C18) was applied as a sorbent. The sorbent was conditioned with methanol (3 mL), 2 % acetic acid in acetonitrile (3 mL) and 2 % acetic acid in water (3 mL). The analytes were eluted by means of (in sequence) methanol (3 mL), ethyl acetate (2 mL) and 0.2 % acetic acid in acetonitrile (2 mL). The same extraction procedure was applied for sediment samples. The values of analytes recoveries from sediment samples were comparable to those obtained from soil samples. From water samples (1 L), triazines were extracted on SDB sorbents. The sorbnet was conditioned with methanol (3 mL), dichloromethane (10 mL), acetonitrile (10 mL) and water (10 mL). The analytes elution was conducted by means of mixture of methanol and ethyl acetate (6 mL, 1:1, *v*/*v*) followed by methanol (5 mL) and acetonitrile (5 mL). The detailed parameters of these extraction procedures are placed in Barchanska et al. (2012).

The mobile phase for triazines separation consisted of 0.05 % TFA in water (A), water (B) and 0.05 % TFA in acetonitrile (C). The gradient profile during separation was as follows: 0 min: 95 % A, 5 % C, flow: 1 mL/min; 2 min: 95 % A, 5 % C, flow: 1 mL/min; 5 min: 75 % A, 25 % C, flow: 1 mL/min; 8 min: 75 % B, 25 % C, flow: 1 mL/min; 12 min: 50 % B, 50 % C, flow: 0.5 mL/min; 18 min: 90 %B, 10 % C, flow: 1 mL/min and 25 min: 95 % A, 5 % C, flow: 1 mL/min (Barchanska et al. 2012). Method fortification recoveries for soil and sediment were in the range of 80–97 %, whereas for water samples, 71–97 % depending on the analyte. The limits of detection (LOD) were 2–88 ng/g and 0.04–0.61 μg/L for soil (sediment) and water samples, respectively.

#### Triketones determination

The detailed procedure for triketone extraction from soil and sediment samples is described in Barchanska et al. (2016). Briefly, soil and sediment samples (10 g) were mixed with acetonitrile (30 mL) and shaken for 30 min. Next, the extracts were filtered. The filtrate was extracted with dichloromethane (2 × 5 mL). After phase separation, the organic layer was evaporated to dryness, and the residue was dissolved in 0.1 M HCl (5 mL). The final purification and concentration of the extract was conducted by means of SPE. The obtained solution was transferred on SDB sorbent, previously conditioned with water (3 mL) and methanol (3 mL). The analytes were eluted by means of methanol (2 mL), ethyl acetate (2 mL) and 4 % acetic acid in acetonitrile (3 mL). After elution, the solvents were evaporated to dryness and the residue was dissolved in 0.5 mL of methanol before chromatographic analyses.

Triketones from water samples were extracted according to the following procedure: a water sample (250 mL) was aspirated through the HLB sorbent, previously conditioned with methanol (6 mL) and water (6 mL) Analytes were eluted by means of the mixture acetonitrile/methanol (6 mL, 1:1, *v*/*v*). After analyte elution, the extract was evaporated to dryness under a stream of nitrogen and the residue was dissolved in 1 mL of methanol. The detailed validation data of this procedure are available from Barchanska et al. (2014).

The mobile phase used for triketones separation consisted of 0.05 % TFA in water (A) and acetonitrile (B). The gradient profile during separation was as follows: 0 min: 100 % A, flow: 0.7 mL/min; 28 min: 60 % A, 40 % B, flow: 0.7 mL/min and 35 min: 30 % A, 70 % B, flow: 1.0 mL/min. Barchanska et al. (2014).

The recoveries of triketone herbicides from soil were in the range of 67–107 %, whereas for sediment were in the range of 78–98 %. LOD was 4–72 ng/g and 5–60 ng/g for soil and sediment, respectively. The triketones recoveries from water samples were in the range of 52 to 96 %, whereas LOD was in the range of 0.06–0.26 μg/L.

The chromatograms of pesticide free and spiked with standards sediment, soils and water samples are presented in Figs. 1SM, 2SM and 3SM, respectively, in Supplementary Material.

To identify target compounds, retention times and UV spectra of sample components and standards were compared as well as standards addition to sample extract was conducted. Moreover, in ambiguous cases, the derivative spectrophotometry was applied to confirm the purity of the chromatogram peak (data not shown). The detailed validation parameters are placed in Table 3. Intra-day precision (repeatability) is referred as within-day precision, whereas inter-day precision is referred to between-day precision. In all cases for the quantitative analysis, the peak area was applied.

**Table 3:** Quality parameters of the method

Soil						
Analyte	Equation	*R* ^2^	LOD [ng/g]	Recovery [%]	Intra-day [CV %] 0.05 μg/g (5 μg/g)	Inter-day [CV %] 0.05 μg/g (5 μg/g)
ATR	*y* = 8.60 × 10^5^ *x −* 3.50 × 10^5^	0.9989	0.02	85 ± 3	6.7 (5.1)	7.0 (5.8)
DEDIA	*y* = 1.46 × 10^4^ *x* + 4.00 × 10^3^	0.9998	0.88	91 ± 4	7.0 (6.2)	7.7 (6.8)
DEA	*y* = 3.60 × 10^5^ *x* − 4.79 × 10^4^	0.9995	0.05	80 ± 3	5.8 (4.7)	5.8 (4.9)
DIA	*y* = 3.90 × 10^5^ *x* + 3.95 × 10^4^	0.9985	0.04	97 ± 7	6.0 (4.9)	6.8 (5.8)
HA	*y* = 1.97 × 10^5^ *x* − 1.98 × 10^4^	0.9972	0.05	86 ± 5	6.6 (6.3)	6.6 (6.4)
MES	*y* = 21.88 × 10^4^ *x* − 40.71 × 10^4^	0.9979	22	106 ± 7	5.6 (4.7)	6.2 (5.8)
AMBA	*y* = 74.09 × 10^4^ *x* − 10.21 × 10^4^	0.9935	4	76 ± 4	5.8 (3.7)	6.4 (6.0)
MNBA	*y* = 35.62 × 10^4^ *x* − 38.12 × 10^4^	0.9994	5	75 ± 2	7.8 (5.9)	8.3 (7.5)
SUL	*y* = 10.35 × 10^4^ *x* + 9.86 × 10^4^	0.9958	15	107 ± 4	6.9 (5.1)	7.3 (6.9)
CMBA	*y* = 13.76 × 10^4^ *x* + 33.11 × 10^4^	0.9977	72	67 ± 12	7.0 (4.5)	7.0 (6.8)
Sediment						
Analyte	Equation	*R* ^2^	LOD [ng/g]	Recovery [%]	Intra-day [CV %] 0.05 μg/g (5 μg/g)	Inter-day [CV %] 0.05 μg/g (5 μg/g)
ATR	*y* = 8.80 × 10^5^ *x* − 3.90 × 10^5^	0.9999	0.03	87 ± 4	6.9 (5.3)	7.2 (6.0)
DEDIA	*y* = 1.50 × 10^4^ *x* + 4.28 × 10^3^	0.9998	0.85	89 ± 3	7.3 (6.6)	8.0 (7.5)
DEA	*y* = 3.60 × 10^5^ *x* − 4.81 × 10^4^	0.9998	0.04	88 ± 6	5.5 (4.1)	5.4 (4.2)
DIA	*y* = 4.00 × 10^5^ *x* + 3.9 × 10^4^	0.9979	0.09	95 ± 5	5.8 (4.5)	6.0 (5.0)
HA	*y* = 1.81 × 10^5^ *x* − 1.56 × 10^4^	0.9998	0.05	87 ± 7	6.5 (6.8)	6.6 (6.5)
MES	*y* = 24.57 × 10^4^ *x* − 11.90 × 10^4^	0.9974	20	89 ± 9	5.9 (5.0)	6.8 (5.8)
AMBA	*y* = 35.67 × 10^4^ *x* − 20.44 × 10^4^	0.9949	5	78 ± 7	5.8 (4.2)	7.0 (6.6)
MNBA	*y* = 22.96 × 10^4^ *x* − 9.55 × 10^4^	0.9991	5	85 ± 9	7.9 (6.0)	8.7 (7.9)
SUL	*y* = 9.63 × 10^4^ *x* + 62.18 × 10^4^	0.9989	60	98 ± 9	6.7 (5.5)	7.0 (6.8)
CMBA	*y* = 65.12 × 10^4^ *x* + 74.88 × 10^4^	0.9988	20	91 ± 12	7.3 (4.9)	7.6 (7.0)
Water						
Analyte	Equation	*R* ^2^	LOD [μg/L]	Recovery [%]	Intra-day [CV %] 10 μg/L (200 μg/L)	Inter-day [CV %] 10 μg/L (200 μg/L)
ATR	*y* = 1.16 × 10^6^ *x* + 5.26 × 10^6^	0.9998	0.35	92 ± 2.1	2.9 (1.2)	3.3 (1.8)
DEDIA	*y* = 1.67 × 10^4^ *x* − 2.84 × 10^4^	0.9995	0.61	87 ± 3.2	4.1 (3.5)	4.1 (3.6)
DEA	*y* = 9.24 × 10^4^ *x* + 5.78 × 10^3^	0.9998	0.19	74 ± 3.6	2.5 (1.4)	2.9 (1.7)
DIA	*y* = 8.47 × 10^5^ *x* − 2.48 × 10^5^	0.9998	0.04	84 ± 5.1	3.1 (2.3)	3.8 (2.8)
HA	*y* = 2.16 × 10^5^ *x* − 1.05 × 10^4^	0.9997	0.14	86 ± 4.2	3.8 (2.7)	4.0 (2.9)
MES	*y* = 2.22 × 10^6^ *x* + 3.26 × 10^5^	0.9988	0.12	87 ± 3.4	3.0 (2.2)	3.8 (2.5)
AMBA	*y* = 1.11 × 10^5^ *x* + 3.37 × 10^5^	0.9998	0.06	96 ± 2.8	3.1 (2.3)	3.1 (2.5)
MNBA	*y* = 4.06 × 10^5^ *x* + 4.81 × 10^5^	0.9999	0.15	52 ± 3.0	2.1 (2.0)	2.8 (2.0)
SUL	*y* = 3.64 × 10^6^ *x* + 1.26 × 10^3^	0.9997	0.20	80 ± 2.7	1.9 (1.5)	2.1 (1.5)
CMBA	*y* = 3.81 × 10^6^ *x* + 2.88 × 10^4^	0.9998	0.26	76 ± 3.5	3.2 (2.7)	3.8 (2.2)

*n* = 9, *μ* = 0.05; analytical wavelength: ATR, DEDIA, DEA, DIA: 220 nm; HA: 240 nm; MES: 230 nm; AMBA: 225 nm; MNBA: 222 nm; SUL: 240 nm; CMBA: 222 nm

#### Organic carbon content and pH

Since pH and organic carbon content (OC) have an influence on pesticides stability in soil and sediment, these parameters were determined according to PN-ISO 10390: 2007 and PN-ISO 14235 (1997). All calculations and the final results are expressed per 1 g of air-dried soil (sediment).

## Results

**Fig. 2:**
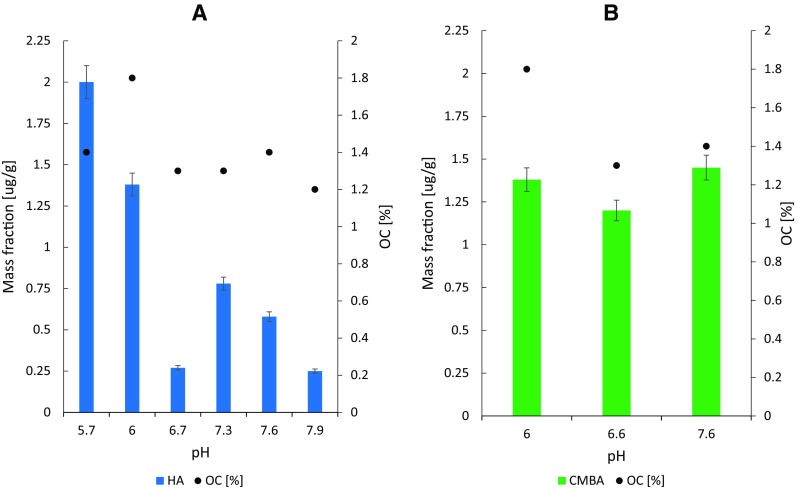
Concentration of HA (**a**) and CMBA (**b**) in sediment samples

Among all investigated herbicides and their degradation products, two degradation products of ATR, HA and DIA; and one degradation product of SUL, CMBA, were detected in sediment samples. The concentration of HA and CMBA correlated to the OC and pH of particular sediments were presented in Fig. 2 The concentration of DIA was 1.1 μg/g.

**Fig. 3:**
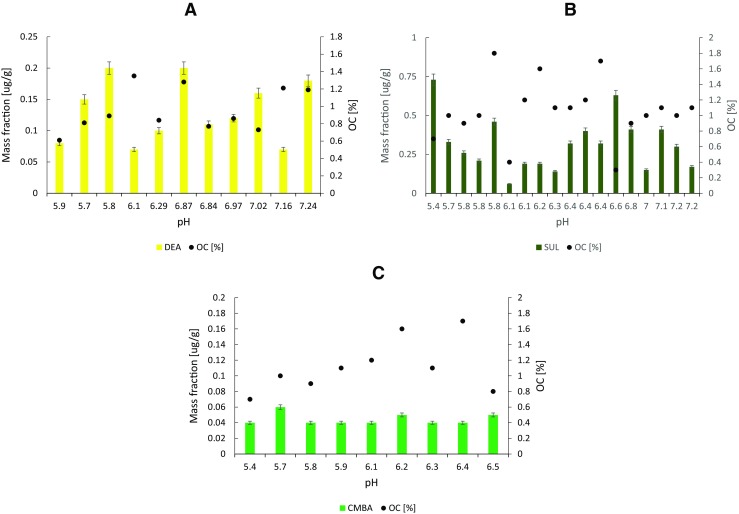
Concentration of DEA (**a**), SUL (**b**) and CMBA (**c**) in soil samples

In the collected soil samples, atrazine degradation products DEA and DIA, as well as SUL and its degradation product CMBA were found. The results of DEA, SUL and CMBA determination in soil samples are presented in Fig. 3. DIA was determined in four samples in the concentration range of 0.04–1.64 μg/g.

**Fig. 4:**
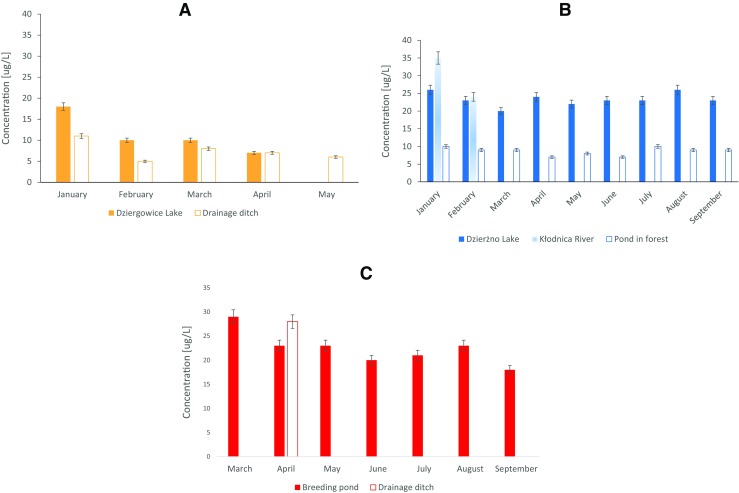
The concentration of DIA (**a**), HA (**b**) and AMBA (**c**) in surface water samples

The results of atrazine, triketones and their degradation products determination in collected surface water samples were presented in Fig. 4.

Moreover, MNBA was found in water form drainage ditch at concentration of 147 μg/L (sample collected in April), and SUL and CMBA were found in water from the Kłodnica River (sample collected in March). The concentrations of analytes were 57 (SD = 5) and 143 (SD = 2) μg/L, for SUL and CMBA, respectively.

### Chemometric analysis

**Fig. 5:**
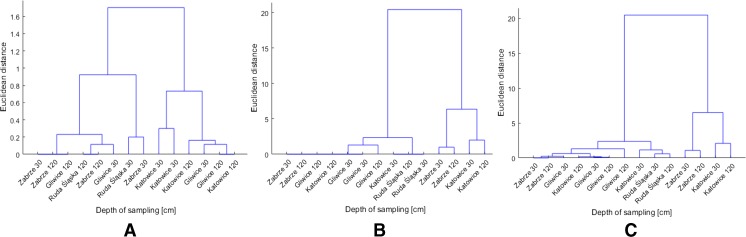
Dendrograms of pH (**a**), OC content (**b**) and for OC content and pH (**c**) of sediments samples collected at 30- and 120-cm depth

Preliminary analysis of dendrograms obtained by means of CA showed no significant differences in sediments’ pH at varying sampling depth. The dendogram of sediments’ pH for samples collected at 30 and 120 cm depth is presented in Fig. 5a.

In all four sampling areas (Zabrze, Katowice, Ruda Slaska, Gliwice) pH of sediments is comparable (Fig. 5a). The OC content in sediments was also subjected to CA analysis (Fig. 5b). The simultaneous CA analysis of OC content and pH showed strong similarity between samples from one place regardless of the sampling depth, what is presented in Fig. 5c.

**Fig. 6:**
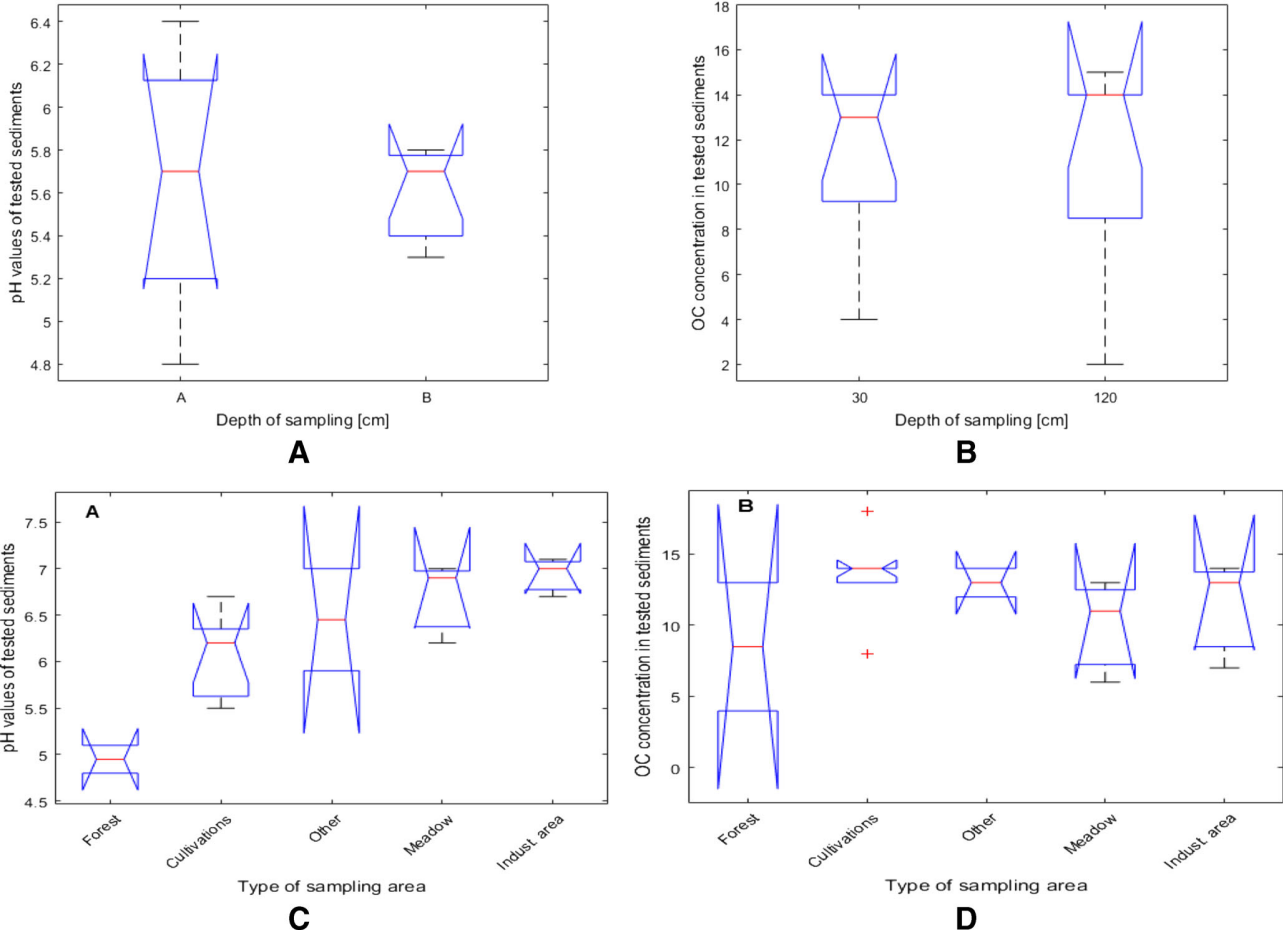
Diagram of between-groups variation for pH values (**a**) and OC content (**b**) of sediment samples. Diagram of between-groups variation of pH values in sediment samples depending on the type of sampling area (**c**). Diagram of between-groups variation of OC content in sediments depending on sampling area (**d**)

Relationships resulting from the dendrogram analysis were confirmed by single and multi-dimensional variance analysis. In case of the influence of the sampling depth on sediments and pH, the calculated *p* value is equal to 0.763 (see Fig. 6 and Table 4). The mean pH of sediments collected from 30-cm depth is not significantly different from the mean pH of samples collected at 120-cm depth (Fig. 6a).

**Table 4:** ANOVA table

Source	SS	Df	MS	*F*	Prob > *F*
Table for pH values of sediment samples					
Groups	0.018	1	0.018	0.095	0.763
Error	2.246	12	0.187	–	–
Total	2.264	13	–	–	–
Table for OC content in sediment samples					
Groups	0.071	1	0.071	0	0.953
Error	235.143	12	19.595	–	–
Total	235.214	13	–	–	–
Table for pH values in sediment samples					
Groups	1.958	7	0.280	5.5	0.027
Error	0.305	6	0.051	–	–
Total	2.264	13	–	–	–
Table for OC content in sediment samples depending on type of sampling area					
Groups	53.590	4	13.397	1.080	0.409
Error	148.881	12	12.407	–	–
Total	202.471	16	–	–	–

*source* the source of the variability; *SS* the sum of squares due to each source; *df* the degrees of freedom associated with each source; *N* the total number of observation; *k* the number of groups; *N-k* within-groups degrees of freedom (error), *k* − 1 the between-groups degrees of freedom (columns); *N* the total degrees of freedom. *N* − 1 = (*N* − *k*) + (*k* − 1); *MS* the mean squares for each source, which is the ratio SS/df; *F F* statistic, which is the ratio of the mean squares; *Prob > F* the *p* value, which is the probability that the *F* statistic can take a value larger than the computed test-statistic value. ANOVA derives this probability from the cumulative distribution function of *F* distribution; *Groups* variability due to the differences among the group means (variability between groups); *Error* variability due to the differences between the data in each group and the group mean (variability within groups); *Total* total variability

In case of OC content, calculated *p* value was 0.953 (see Fig. 6b and Table 4) and, as in the case of the pH, there was no significant difference between studied groups.

Sediment samples were divided into five groups according to the area where they were collected:

1. Forest
2. Cultivations
3. Meadow
4. Industrial area
5. Other

Such grouping of sediment samples allowed verifying the thesis, that the area from which samples were collected had an influence on the analysed variables. For this purpose, a one-way analysis of variance was applied. Fig. 6c shows the differences in pH of sediment samples depending on the type of sampling area.

Based on *p* value, it was concluded, that the difference between average pH for studied groups is statistically significant—(*p* value =0.003), what is presented in Table 4).

The differences in OC in samples from different sampling areas were not statistically different, as is presented in Fig. 6d and in Table 4.

Chemometric analysis was also applied to the results obtained from the soil samples. In neither of the studied cases was a statistically significant relationship between the concentrations of examined herbicides, their degradation products and soil parameters (OC, pH) was observed. The *p* values were as follows:

- *p* value_pH = 0.2373
- *p* value_OC = 0.3439
- *p* value_ATR = 0.3535
- *p* value_MES = 0.5855
- *p* value_SUL = 0.2327

## Discussion

### Triazines

Stability of triazines and their degradation products has attracted scientific attention because these compounds are present in environment after over 10 years after the withdrawal from the use (Garbin et al. 2007; Prosen et al. 2007; Hutta et al. 2011; Li et al. 2012). This is the consequence of the long-term transfer of residues from agricultural areas where these herbicides were previously used.

Atrazine fate in the environment, e.g. sorption, leaching and degradation depends, inter alia, on soil characteristics as well as environmental conditions; therefore, the prediction of their stability is a difficult task.

FTIR, differential thermal analysis (DTA) and ^1^H-NMR studies on interactions between atrazine and other s-triazine compounds with humic substances suggested the occurrence of H-bonds, possibly involving carbonyl groups of humic acids and secondary amine groups of the s-triazine. It was found that the adsorption capacity for the s-triazines is related to humic substances content and titratable acidity of the soil (Nearpass 1976). It was inferred that adsorption occurred by H-bonding between the amino protons of the triazine ring and humic acids. The positive correlation between the *K*_oc_ and octanol-water partition coefficient values may implicate that although hydrogen-bonding is important in triazine-soil organic matter interactions (SOM), this type of complexation is probably governed by hydrophobic partitioning-like interactions. Similar conclusions were reached by Kulikova and Perminova (2002). According to their investigations, triazinic compounds are sorbed by sorbents containing a high level of aromatic structures, therefore, hydrophobic bonding is responsible for the interactions of these compounds with soil organic matter. On the other hand, Chefetz (2003; Chefetz et al. 2004), and Salloum et al. (2002; Alletto et al. 2010) claimed, that atrazine exhibits also affinity to aliphatic domains of soil organic matter.

ATR was not detected in the sediment, soil or water samples. Since it has not been used for many years, it is degraded in the environment, and its concentration is below limit of quantification (LOQ) of the applied analytical method. However, other researchers (i.e. Farlin et al. 2013, Geng et al. 2013) detected ATR in soil samples at concentrations up to 11.1 μg/kg. Since the stability of pesticides is strongly influenced by soil texture, organic matter content and type and activity of microorganisms, the direct comparison of this type research obtained by different research groups is difficult.

DEA was detected in 11 soil samples (sample nos. 21, 22, 24, 26–29, 30, 31, 33, 34) in the concentration range of 0.07–0.18 μg/g (Fig. 2). No relationship between the concentration of this compound and soil pH nor OC was observed. In soils that are characterized by low pH, strong interaction between DEA and soil occurs that hinder this pesticide’s elution by surface water and its migration to sediments. Therefore, the absence of DEA in sediment and water samples was observed.

DIA was detected in sediment from river Bobrówka (sample no. 12) at a concentration of 1.1 μg/g (SD = 0.1 μg/g) and soil samples from Szczekociny I (sample no. 28), Sucha Beskidzka (sample no. 22), Tychy Wilkowyje II (no. 26), and Rudziniec (no. 39) in the range of 0.04–1.64 μg/g.

The presence of DIA in the surface water from the drainage ditch in Rudziniec was the result of its presence in the soil (sample no. 39, DIA concentration 1.50 (SD = 0.25) μg/g) from the same location. This compound was transported to surface water with atmospheric precipitation; therefore, its concentration was strictly related to rainfall, snowfall and its thaw. DIA concentration in water from this location varied form 5 to 11 μg/L in the period January–June. In July and August, it was also detected; however, its concentration was below limit of quantification (LOQ), due to scanty rainfall. DIA was also detected in water from Dziergowice Lake in the concentration range of 7–18 μg/L in the period January–April.

Caquet et al. (2013) reported both DEA and DIA presence in estuarine continuums in the Bay of Vilaine area (France). However, the concentration of these compounds was significantly lower than in the present report (0.89 and 0.55 μg/L for DEA and DIA, respectively). This discrepancy was caused by different place of sampling: agricultural area versus estuary area, where the concentration of pesticides is lower because of a much larger mass of water in comparison to local streams and local lakes. Moreover, in France atrazine is forbidden since 2003, whereas in Poland since 2008.

HA was determined in six sediment samples (sample nos. 2, 11, 12, 14, 16, 17), its concentration was in the range of 0.25–2.00 μg/g. HA was detected in ten soil samples; however, its concentration was below the LOQ of the applied analytical method. The presence of HA in sediment samples was caused by its relatively low water solubility and the longest half-life among all investigated triazine compounds (Table 1); therefore, it accumulated in sediment. It should be noted that HA was detected in regions where mainly maize has been cultivated and in the vicinity of a water treatment plant. The results indicated the high stability of this ATR degradation product and its tendency to accumulate in sediments, as well as its persistence in the processes of water purification. Moreover, a negative correlation between the HA content and sediment pH was observed. Probably, HA sorbed stronger on the mineral components of soil, rather than on organic matter. HA was detected in water samples collected form Dzierżno Lake, Kłodnica River and a pond surrounded by forest. Its concentration in the stagnant water reservoir was stable during the entire experimental period, and was in the range of 20–26 and 7–10 μg/L from water from Dzierżno Lake and a pond in a forest, respectively. HA was determined in water from Kłodnica River only in January and February (concentration range 24–35 μg/L). In the remaining months, it was also detected but its concentration was below the LOQ.

Geng et al. (2013) determined ATR and its degradation products in soil and water samples collected in one of the agricultural area in China, where atrazine is still used. According to their studies, the mean concentration of ATR was 106.8 ng/L and 11.1 μg/kg; DEA was 0.9 ng/L and 0.4 μg/kg and HA was 0.3 ng/L and 7.8 μg/kg for soil and water samples, respectively. The comparison of these results with the results presented in this work indicates a gradual decrease in the concentrations of atrazine and its derivatives, in the regions in which it was withdrawn from use. However, this loss is not for each compound the same. This is due to the complex degraded processes in the environment.

### Triketones

In the collected soil and sediment samples neither MES nor its degradation products were detected, since these compounds are well-soluble in water. Moreover, AMBA and MNBA are characterized by the shortest half-life in soil among all investigated triketone by-products (Table 1).

CMBA, the degradation product of SUL, was determined in sediment samples. It was present in three samples (3; 12; 16), in the concentration range of 1.20–1.45 μg/g. These samples were collected in the vicinity of maize cultivation and water treatment plants.

In case of sample nos. 2 and 3, where HA and CMBA were detected, respectively, both analytes were present in the upper layer of the sediment. It may suggest that these compounds are strongly bound to the sediment particles that preclude their migration into deeper layers of sediment.

SUL was determined in 18 soil samples (18–20; 22–25; 27–30; 34–40) in the concentration range of 0.06–0.73 μg/g. No correlation between OC nor pH and SUL content in soil was observed. CMBA, the SUL degradation product, was determined in nine soil samples (18; 22; 24; 27–29; 37; 38; 40) in the concentration range of 0.04–0.06 μg/g. CMBA coexisted with SUL, but only in samples that pH was below 6.5. SUL was determined in soil samples collected from the maize and potatoes cultivation (that is applied as a crop rotation with maize).

SUL and CMBA were determined in water from Kłodnica River, but only in March—the month of intense agrotechnical intervention. Their concentration was high (57 and 143 μg/L, for SUL and CMBA, respectively), however, transient.

AMBA, MES degradation product, was determined in water collected from the breeding pond. The maximum concentration of AMBA (29 μg/L) was determined in March, and then it was slowly decreased to 18 μg/L at 6 months. This slow decrease in the AMBA concentration was due to the slow exchange of water between the pond and its tributaries and the highest stability of this compound in environment among all investigated triketones and their degradation products (Table 1; Barchanska et al. 2016). MNBA was determined in water from the drainage ditch at a concentration of 147 μg/L (sample collected in April).

The obtained data could not be compared with regulatory AA-EQS and MAC-EQS for priority substances, because triketones and their degradation products and ATR degradation products are not included in these regulations (EU 2008). However, on the basis of the presented study, the concentration of triketone herbicides and their metabolites as well as ATR degradation products should be monitored, due to their stability in the environment.

## Conclusion

Seven years after the withdrawal from use in Poland, ATR was not detected in any of the collected samples. However, its degradation products are still present in environment.

Forty-one percent of sediment, 71 % of soil and 8 % of surface water samples contained these compounds. DIA was the most common ATR degradation product found. It was determined in soil (0.04–1.62 μg/g), sediment (1.1 μg/g) and water (5–18 μg/L) samples.

Among triketone herbicides, SUL was the most common compound found, and it was determined in 85 % of soil samples (0.06–0.73 μg/g); its degradation product (CMBA) was present in 43 % of soil samples and in 17 % of sediment samples. Sediments and soil samples were free of MES and its degradation products.

MNBA and AMBA as well as SUL and CMBA were detected occasionally in surface water samples. Although their concentration was high (up to 147 μg/L for MNBA), it was transient, highly influenced by rainfalls.

The results obtained for triketones may be indicative of their rapid degradation in environment. A statistically significant relationship was not found between the concentrations of examined herbicides, their degradation products and soil parameters (OC, pH).

The half-life of herbicides in the environment given by the producers of agrochemicals does not always correspond to their actual persistence in the environment. Pesticide stability in the environment is influenced by many factors (temperature, composition and pH of soil (sediment), type and activity of microorganisms, etc.) that are difficult to predict under experimental conditions.

## Electronic supplementary material

ESM 1 (DOCX 644 kb)
