# Supplementary material for: Atrazine, triketone herbicides, and their degradation products in sediment, soil and surface water samples in Poland
Source: Environ Sci Pollut Res Int. 2016 Oct 14;24(1):644–58. doi: 10.1007/s11356-016-7798-3 (PMC5219039; doi:10.1007/s11356-016-7798-3)
Supplement: Supplementary file 1 — (DOCX 644 kb) [file 11356_2016_7798_MOESM1_ESM.docx]

Hanna Barchanska^1^, Marcin Sajdak^2^, Kornelia Szczypka^1^, Angelika Swientek^1^, Martyna Tworek^1^, Magdalena Kurek^1^

- **Atrazine, triketone herbicides, and their degradation products
  in sediment, soil and surface water samples in Poland**
- ^1^ - Department of Inorganic, Analytical Chemistry and Electrochemistry, Faculty of Chemistry, Silesian University of Technology, B. Krzywoustego 6 Str, 44-100, Gliwice, Poland
- ^2^ – Institute for Chemical Processing of Coal, 1 Zamkowa St. 41-803 Zabrze, Poland,

* - corresponding author; e-mail: [hanna.barchanska@polsl.pl](mailto:hanna.barchanska@polsl.pl); tel: +48 32 237 28 18; fax: +48 32 237 12 05


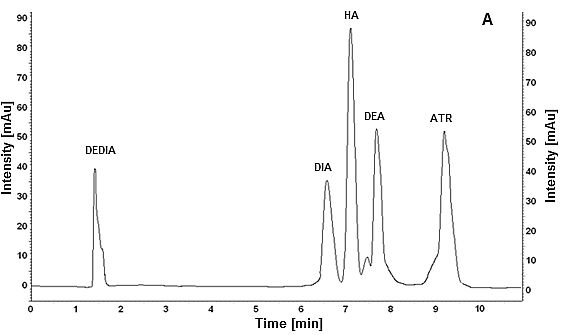


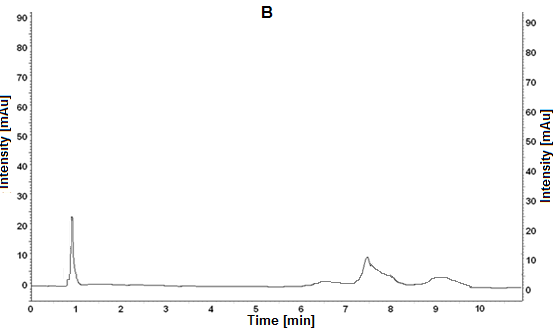


**B**

**A**


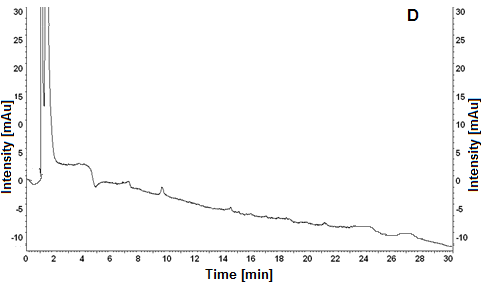


**C**

**D**

**
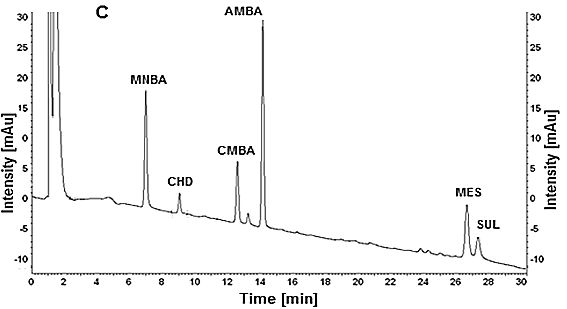
**

**Fig. 1SM** Chromatogram of extracts of blank (Fig.1A and Fig.1D) and spiked (Fig.1B and Fig.1C) sediment sample (concentration 1 µg/g; λ=230 nm)


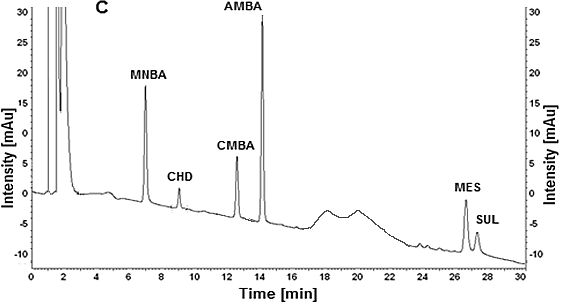

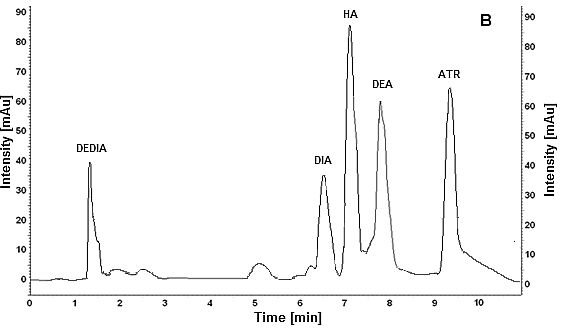
**Fig.2 SM** Chromatogram of extracts of blank (Fig.2A and Fig.2D) and spiked (Fig.2B and Fig.2C)

**A**


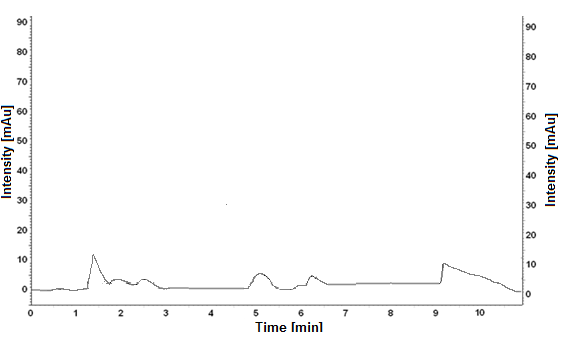

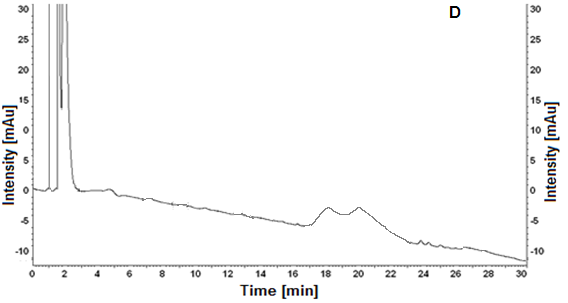

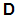


soil sample (concentration 1 µg/g; λ=230 nm)

**
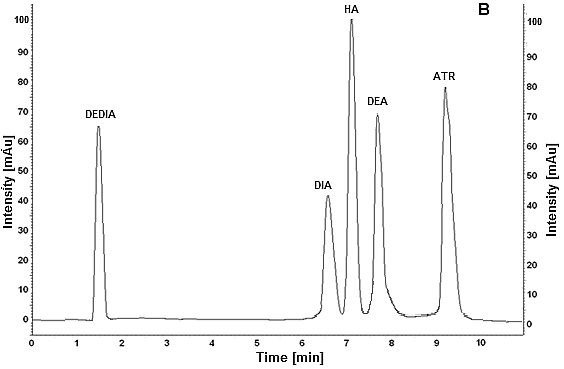
`**


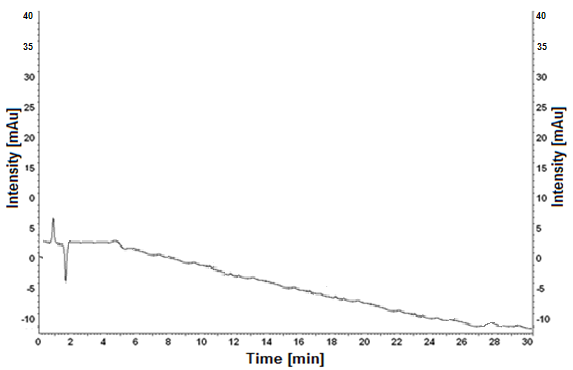

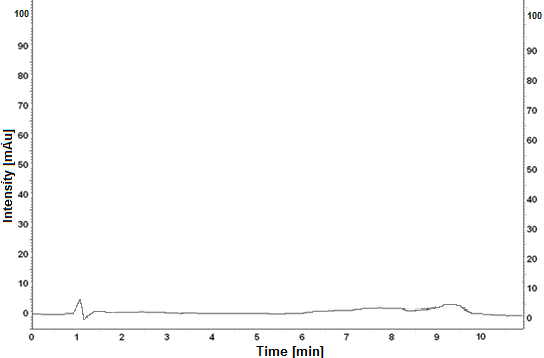


**A**

**B**

**D**

**
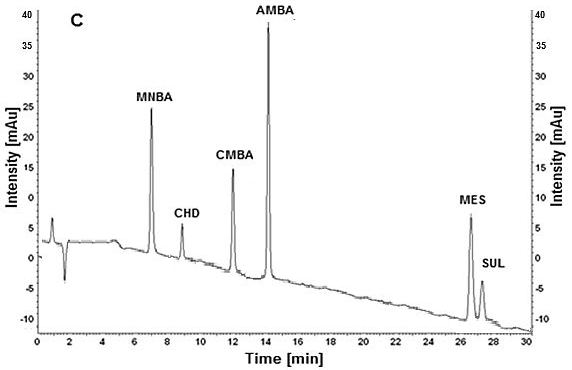
**

**Fig. 3SM** Chromatogram of extracts of blank (Fig.3A and Fig.3D) and spiked (Fig.3B and Fig.3C) water sample (concentration 10 µg/mL; λ=230 nm)
